# Reviewer acknowledgement 2013

**DOI:** 10.1186/1471-213X-14-1

**Published:** 2014-01-16

**Authors:** Philippa K Harris

**Affiliations:** 1 BioMed Central, 236 Gray's Inn Road, London WC1X 8HB, UK

## Abstract

**Contributing reviewers:**

The editors of *BMC Developmental Biology* would like to thank all our reviewers who have contributed to the journal in Volume 13 (2013).

## 

Amir Abbasi

Pakistan

Aziz Aboobaker

UK

Takashi Adachi-Yamada

Japan

Curie Ahn

South Korea

Michael Akam

UK

Denise Al Alam

USA

Declan W. Ali

Canada

Christopher Antos

Germany

Hiroshi Asahara

Japan

Brandon Atkins

USA

Michalis Averof

France

Amy Bejsovec

USA

Philip Benfey

USA

Sophie Berland

France

Ira Blitz

USA

Gareth Bloomfield

UK

Laure Bonnaud

France

Ewa Borsuk

Poland

Derek Brazil

UK

Eric Brunskill

USA

Annalisa Buffo

Italy

Kenneth Cadigan

USA

Michel Cayouette

Canada

Francesc Cebria

Spain

Helen Chamberlin

USA

Gavin Chapman

Australia

Karen Chapman

UK

Tatiana Chebotareva

UK

Chiann-Mun Chen

UK

Xin Chen

USA

Yiping Chen

USA

Marta Chiodin

Spain

Ariel Chipman

Israel

Marcelle Christophe

Australia

Vincenzo Cirulli

USA

Wim Damen

Germany

Nicolas David

France

Fiorenza De Bernardi

Italy

Jose De Celis

Spain

Jose Luís De La Pompa

Spain

Arnold De Loof

Belgium

Bernie Degnan

Australia

Mary Donohoe

USA

Gregg Duester

USA

Bertrand Duvillie

France

Kai Erdmann

UK

Janice Evans

USA

Alyson Fournier

Canada

Paul Francois

Canada

Alison Frand

USA

Tamara Franz-Odendaal

Canada

Elaine Fuchs

USA

John Gerhart

USA

James Godwin

Australia

Mike Golding

USA

Kaoru Goto

Japan

Valerie Gouon-Evans

USA

Daniel Graf

Greece

Francois Graner

France

Tina L. Gumienny

USA

Mukesh Kumar Gupta

India

Benedikt Hallgrimsson

Canada

Alexandra Harvey

Australia

Toshinori Hayashi

Japan

Andreas Hejnol

Norway

Clarissa Henry

USA

Kathryn Hentges

UK

Wiebke Herzog

Germany

Andreas Heyland

Canada

Ben Hogan

Australia

Bernadette Holdener

USA

Ruijin Huang

Germany

Wen Huang

USA

Shuntaro Ikeda

Japan

Kei Inouye

Japan

Naoki Iwamori

Japan

Fari Izadyar

USA

Ralf Janssen

Sweden

Rulang Jiang

USA

Rosalind John

UK

Robert Kelly

France

Eunjoon Kim

South Korea

Heinz Köhler

Germany

Tetsuro Kokubo

Japan

Michael Kuehl

Germany

Ewart Kuijk

Netherlands

Nicholas Lakin

UK

Pierre Leclerc

Canada

Hojoon Lee

USA

Mark Leid

USA

Baojie Li

Singapore

Baojie Li

China

Shaohua Li

USA

Ching-Ling (Ellen) Lien

USA

Gufa Lin

USA

Jerry Lingrel

USA

Claudia Linker

UK

Jun Liu

USA

Jining Lu

USA

Carmel Mcdougall

Australia

Ralph Marcucio

USA

Osvaldo Marinotti

USA

Nirmala Mavila

USA

Carmel Mcdougall

Australia

Andreas Meinhardt

Germany

Isabel Merida

Spain

Martin Meyer

UK

Frederic Michon

Finland

Carolina Minguillon

Spain

Satomi Miyagawa

UK

Jamileh Movassat

France

Alex Nechiporuk

USA

Kenichi Nishioka

Japan

Danton O'Day

Canada

Leif Oxburgh

USA

Nancy Papalopulu

UK

David Parichy

USA

Ketan Patel

UK

Catherine Pears

UK

Bret Pearson

Canada

Roberta Pennati

Italy

Laura Perin

USA

Christian Petersen

USA

Berenika Anna Plusa

UK

Patricia Purcell

USA

Tamar Resnick

USA

Gustavo Rezende

Brazil

Sandra Rieger

USA

Jason Rock

USA

Bernard Roelen

Netherlands

Stéphane Roy

Canada

Michael Rudnicki

Canada

Charles Sagerstrom

USA

Masao Sakai

Japan

Daniel Salamone

Argentina

Guillaume Salbreux

Germany

Hiroshi Sasaki

Japan

Ken Sawai

Japan

Thomas Schilling

USA

Axel Schweickert

Germany

Guy Smagghe

Belgium

Barbara Stay

USA

Gerhard Steiner

Austria

Gang Sun

China

Qing-Yuan Sun

China

Makoto Tachibana

Japan

Hiroyuki Takeda

Japan

Jun K. Takeuchi

Japan

Mikiko Tanaka

Japan

Andrzej Tarkowski

Poland

Fabrice Teletchea

France

Carsten Theiss

Germany

Bernhard Tins

UK

Matthew Topham

USA

Eric Turner

USA

Alexander Vaiserman

Ukraine

Srinivasan Vijayaraghavan

USA

Bo Wang

USA

Shugo Watabe

Japan

Gunther Wennemuth

Germany

Christopher West

USA

Elspeth Whitby

UK

Joost Woltering

Switzerland

Ji Wu

China

Zhongzhou Yang

China

Laura Yates

Australia

Pamela Yelick

USA

Hitoshi Yokoyama

Japan

Minoru Yoshida

Japan

Stephane Zaffran

France

Meijia Zhang

China

Xueliang Zhu

China

